# Nematicide efficacy at managing *Meloidogyne arenaria* and non-target effects on free-living nematodes in peanut production

**DOI:** 10.21307/jofnem-2020-028

**Published:** 2020-04-16

**Authors:** Zane J. Grabau, Mark D. Mauldin, Alemayehu Habteweld, Ethan T. Carter

**Affiliations:** 1 Entomology and Nematology Department, University of Florida, 1881 Natural Area Drive, Gainesville, FL 32611; 2 Washington County Extension, University of Florida, 1424 Jackson Ave., Ste A Chipley, FL 32428; 3 Jackson County Extension, University of Florida, 2741 Penn Ave., Ste #3 Marianna, FL 32448

**Keywords:** 1,3-dichloropropene, aldicarb, *Arachis hypogea*, fluopyram, management, *Meloidogyne arenaria*, nematode community, nematicide, peanut, peanut root-knot nematode

## Abstract

*Meloidogyne arenaria* (peanut root-knot nematode (PRKN)) is a major pest of peanut. Nematicide application is an important tool for the management of PRKN. Nematicides with minimal effects on free-living nematodes are desired. Fluopyram nematicide is recently introduced in peanut production and needs to be assessed. The objective of this research is to evaluate fluopyram and the established nematicides 1,3-Dichloropropene (1,3-D) and aldicarb for efficacy at managing PRKN and impacts on free-living nematodes. Nematicides were evaluated in field studies in 2017 and 2018 conducted in commercial peanut fields. All nematicides increased peanut yield in 2017 compared with untreated control, but did not affect soil PRKN abundances or root galling. In 2018, PRKN infestation was too low to accurately assess PRKN management by nematicides. Aldicarb and fluopyram did not affect any free-living nematode trophic group or individual genera. In contrast, 1,3-D decreased total fungivore and fungivore genera *Filenchus* and *Aphelenchus* soil abundances, but did not affect bacterivores, omnivore-predators, total herbivores, or any other nematode genera. In summary, 1,3-D, but not aldicarb or fluopyram, had non-target effects on free-living nematodes, particularly fungivores.

Peanut (*Arachis hypogaea*) is an important crop in the United States with 757,000 ha planted in 2018, worth $1.15 billion (NASS-USDA, 2019a, b). Much of the production is concentrated in the Southeast where *Meloidogyne arenaria* (peanut root-knot nematode (PRKN)) can significantly reduce yields with suppression approaching 50% observed in field research (Rodriguez-Kabana and Robertson, 1987; Rodriguez-Kabana et al., 1994a, 1994b). Damage thresholds for this nematode are 1 egg/100 cm^3^, so any detectable level of this nematode presents a risk of damage (McSorley et al., 1992).

Peanut producers rely on crop rotation, resistant cultivars, and nematicide application to manage PRKN. Peanut cultivars (TifNV High O/L, Georgia 14 N, and Tifguard) that are highly resistant to root-knot nematodes are available and derive resistance from the parental cultivar COAN (Holbrook et al., 2008; Branch et al., 2014; Holbrook et al., 2017). These resistant cultivars are not widely adopted as a majority of acreage is planted to “Georgia 06 G,” which is susceptible to root-knot nematodes. Infrequent use of resistant cultivars can be attributed to a combination of lack of seed supply of resistant cultivars; lesser yield potential of resistant cultivars, particularly “Tifguard” and “Georgia 14 N” (Holbrook et al., 2008; Branch et al., 2014); and greater familiarity with “Georgia 06 G.” Crop rotation to a non-host such as cotton (*Gossypium hirsutum*) or bahiagrass (*Paspalum notatum*) or poor host such as corn (*Zea mays*), rather than host crops such as soybean (*Glycine max*), is an effective option for managing root-knot nematodes in peanuts, particularly when a non-host is grown for two or more years (Rodriguez-Kabana et al., 1994a, 1994b; Johnson et al., 1999; Davis and Timper, 2000). In practice, grower adoption of crop rotations that help manage PRKN varies as nematode management is oftentimes a secondary consideration to crop prices, grower equipment, and environmental conditions when choosing a crop rotation.

Nematicide application is an important and widely used tool for nematode management in peanut production. Traditionally, growers have relied on fumigants, primarily 1,3-Dichloropropene (1,3-D), and older carbamate non-fumigants, such as aldicarb and oxamyl. Aldicarb and 1,3-D can help manage PRKN in peanut production, although efficacy varies by year and environmental conditions (Rodriguez-Kabana and Robertson, 1987; Kinloch and Dickson, 1991; Johnson et al., 1999; Kokalis-Burelle et al., 2002). Recently, a newer chemistry, fluopyram, became available in peanut production. Fluopyram is a benzamide, succinate dehydrogenase inhibitor and was originally used as a fungicide. Nematicidal or nematistatic activity of fluopyram against *Meloidgyne incognita* (southern root-knot nematode (SRKN)) has been demonstrated in vitro (Faske and Hurd, 2015; Ji et al., 2019; Oka and Saroya, 2019). Fluopyram has managed SRKN, to varying degrees, on tomato (*Solanum lycopersicum*) in greenhouse tests (Silva et al., 2019; Dahlin et al., 2019), lima beans (*Phaseolus lunatus*) in microplot tests (Jones et al., 2017), carrots (*Daucus carota*) in field trials (Becker et al., 2019), tomatoes in microplots (Dahlin et al., 2019), and tomatoes in field trials (Ji et al., 2019). Relatively little research has been reported on fluopyram in row crop production. Most published research has focused on fluopyram efficacy as a seed treatment against *Heterodera glycines* (soybean cyst nematode) on soybean with mixed results in that system (Kandel et al., 2017; Beeman et al., 2019). Research is needed to evaluate fluopyram efficacy for managing PRKN in peanut production, particularly given the limited number of nematicides available for peanut production.

Increasingly, effect of pesticides on non-target organisms is an important consideration. In particular, free-living nematodes are a major non-target group of concern when nematicides are applied because they are biologically very similar to the target plant-parasitic nematodes, they can contribute to soil productivity (Khan and Kim, 2005; Trap et al., 2016), and they are sensitive indicators of soil food web function (Ferris et al., 2001, 2012). Free-living nematodes can contribute to soil nutrient cycling (Trap et al., 2016; Holajjer et al., 2016) and pest suppression (Khan and Kim, 2005), which in turn may contribute to sustaining productive soil. Many nematicides, including aldicarb (Smolik, 1983; Grabau and Chen, 2016; Grabau et al., 2018) and 1,3-D (Sanchez-Moreno et al., 2010; Timper et al., 2012; Watson and Desaeger, 2019), are known to negatively affect free-living nematodes in various crop systems, but their impacts in peanut production have not been reported.

Compared with older non-fumigants and fumigants, fluopyram has a more narrow toxicity profile, making it less hazardous for human handlers and macrofauna. Non-target effects of fluopyram in some systems have been investigated. In turfgrass, repeated broadcast applications of fluopyram have negative non-target effects on free-living nematodes to a greater degree than other non-fumigant nematicides (Waldo et al., 2019). In strawberry production, preplant fluopyram application through drip irrigation did not affect free-living nematodes, but 1,3-D did (Watson and Desaeger, 2019). Fluopyram application methods and rates vary by crop system, so information on fluopyram effects in row crop production is needed. Direct comparisons of fluopyram, aldicarb, and 1,3-D effects on free-living nematodes in peanut production will help growers determine if a particular nematicide is a better choice for helping maintain these beneficial organisms.

Given this information, the general objective of this research was to identify effective root-knot nematode management options in peanut production that minimize non-target effects on free-living nematodes. Our hypotheses were that nematicides would vary in their target and non-target effects and that free-living nematode trophic groups and individual genera would vary in their sensitivity to nematicide application. The specific objectives of this research were to determine the influence of fluopyram, 1,3-D, and aldicarb on: root-knot nematode management and associated crop damage; and free-living nematodes in commercial peanut production.

## Materials and methods

### Field site and maintenance

Identical trials were conducted at two sites, one in 2017 and another in 2018. Both sites are commercial peanut production fields in Jackson County, FL. Site 1 (2017) was located at 30.903460,−85.089192 and Site 2 (2018) was located at 30.874462,−85.039201. Both sites were Troup sand soil type. Site 1 (2017) had 86% sand, 7% silt, and 7% clay, whereas Site 2 (2018) had 91% sand, 4% silt, and 5% clay. Both sites had a history of cotton, peanut, and cantaloupe production. Various nematicides, including 1,3-D, aldicarb, and fluopyram, have been used at each site in prior years, but no product has been used continuously at either site. Aside from preplant nematicide treatments, each year of the study the site was maintained uniformly with conventional fertilizer and pesticide management according to standard grower practices for the area. The sites were conventionally tilled and irrigated. Both sites were reported by the grower as having root-knot nematodes. Both years, the root-knot nematode susceptible peanut cultivar “Georgia 06 G” was planted in mid-May (Table 1).

**Table 1.** Schedule for data collection and trial maintenance.

Task	2017	2018
Preplant soil samples	10 April (39)	29 March (52)
1,3-D applied	5 May (14)	4 May (16)
Peanuts planted/in-furrow aldicarb and fluopyram applied	19 May	20 May
Midseason soil samples	20 July (62)	26 July (67)
Harvest soil and root samples	19 September (122)	24 September (126)
Peanuts inverted	2 October (135)	15 October (147)
Peanuts combined	5 October (138)	21 October (153)

**Note:** Numbers in parentheses are days before or after planting.

### Experimental design

The trial was a randomized complete block design. Treatments were preplant nematicide applications: untreated control, 1,3-D preplant in-row application, aldicarb granular in-furrow at-planting, and fluopyram liquid in-furrow at-planting. For treatment two, 1,3-D was applied as Telone II (Dow AgroSciences, Indianapolis, IN) at 32.7 l/ha or 38.6 kg active ingredient (a.i.)/ha. In both years, 1,3-D was applied approximately two weeks before planting using a fumigation rig with shanks 30 cm deep and spaced 91 cm apart − placed in rows where seed would be planted using GPS (Table 1). The shank chisel trace was disrupted and sealed using a rolling basket in order to minimize fumigant loss. This application rate and method is a common practice for Florida peanut growers that use 1,3-D. The application rate is less than the maximum labelled rate because of economic considerations. For aldicarb application, AgLogic 15GG (AgLogic Chemical Company, Chapel Hill, NC) was applied at 7.84 kg/ha (1.18 kg a.i./ha) and dispensed in-furrow at planting from hopper boxes via tubing. For fluopyram application, Velum Total (Bayer CropSciences, Research Triangle Park, NC) was applied in-furrow at planting at 1.32 l/ha (0.24 kg fluopyram a.i./ha). Velum Total also contains the insecticide imidacloprid, which was applied at 0.34 kg a.i./ha. Fluopyram and aldicarb were applied at the maximum labeled rates using the methods specified on the label. Treatments were applied in six-row strips, corresponding to planter width across the length of the field (approximately 1,000 m in 2017 and 400 m in 2018) and replicated four times.

### Soil sampling and nematode quantification

A 9.1-m section of the trial was designated as the sampling plots for nematode assessment because it was not feasible to accurately assess nematode abundances across the entire length of each treatment strip. Because of this discrepancy, nematode counts are not intended to accurately reflect populations in the entire field or correlate to trial yield for the entire strip.

Soil nematode abundances were assessed before fumigation, midseason at 62−67 days after planting (DAP), and just before harvest at 122−126 DAP (Table 1). At each sampling instance, 12 cores were collected from the 9.1 m sampling section within each strip. Cores were collected with an Oakfield tube to 30 cm deep and were collected from the four central rows of the strip and 9 cm or less from rows of peanut plants. Samples were bulked and homogenized by strip.

Nematodes were extracted from soil by sucrose-centrifugation (Byrd et al., 1976) and the nematode community (plant-parasitic and free-living nematodes) was quantified morphologically using an inverted light microscope. Nematode abundances by trophic group (herbivores, fungivores, bacterivores, and omnivores plus predators) were calculated and analyzed (Yeates et al., 1993). Abundances of individual genera that were consistently present in most plots were also analyzed. Genera analyzed included the herbivore *Meloidogyne*; fungivores *Filenchus*, *Aphelenchus*, and *Aphelenchoides*; the bacterivores *Cephalobus*, *Eucephalobus*, and *Acrobeles*; and the omnivore *Aporcelaimellus.* Diversity of individual genera and trophic guilds were estimated by calculating Hills N1 index (Neher and Darby, 2006) using individual genera or trophic guilds as inputs, respectively. Trophic guilds are grouping of nematodes with the same feeding type and c-p values, which reflect similar ecological niches (Bongers, 1990; Bongers and Ferris, 1999). The N1 index is easier to interpret than other diversity indices as N1 values estimate the number of abundant groups; nematode genera or trophic guilds in this case.

### *Meloidogyne* molecular identification

At the 2017 trial site, *M. arenaria* was confirmed as the root-knot nematode species present in the field based on molecular identification of root-knot nematode females extracted from peanuts grown in the trial. At the 2018 trial site, a mixture of root-knot nematode species was suspected based on root-knot nematode population trends. For this reason, in the 2018 trial, root-knot nematode speciation was based on root-knot nematode females extracted from coleus (*Plectranthus scutellarioides*), a host of the major root-knot nematodes in the region, grown in field soil from the 2018 trial. The presence of both *M. arenaria* and *Meloidogyne incognita*, for which peanut is a non-host, were detected in the 2018 trial.

For both trials, identification was conducted as follows. Genomic DNA was extracted from six individually picked gravid females from the root samples for mitochondrial haplotyping (Pagan et al., 2015). The intergenic spacer and part of the adjacent large subunit ribosomal RNA gene (lrDNA) were amplified using MORF/MTHIS and TRNAH/MHR106 primers pairs. The sequence polymorphism in lrDNA revealed by restriction pattern following digestion with the restriction enzymes *HinfI* and *MnlI*, and the mitochondrial haplotypes were determined. Finally, the species identities were confirmed using MI-F/MI-R primer set for four of the females and Far/Rar primer set for two of the females for *M. incognita* and *M. arenaria*, respectively (Adam et al., 2007).

### Crop yield and root damage

Galling on roots and pods was assessed at approximately two to four weeks before harvest each year (Table 1). From each sampling plot, five plants were dug and the root systems were washed free of soil. Roots and pods were washed and rated separately for percent coverage of galls from root-knot nematode.

Crop yield was measured from the four central rows for the entire length of each treated strip. Peanut pods were inverted in early October and were harvested three to six days later (Table 1). Yield was measured for each strip by weighing the peanut-collecting truck before and after harvesting each strip using portable platform scales. Because the length of each strip varied, area harvested for each strip was calculated from field length measured with harvester-mounted GPS units in order to calculate yield per hectare.

### Statistical analysis

Crop yield as well as root-knot nematode abundances and damage were analyzed separately for each year because a mixed population of root-knot nematodes was present in 2018. Root-knot nematode soil abundances were analyzed separately by season (preplant, midseason, and harvest). These variables were subject to one-way ANOVA.

All other variables, including trophic group abundances, abundances of individual nematode genera other than root-knot nematodes, and N1 diversity indices were combined between years individually by season. These variables were subject to a modified two-way (year by nematicide treatment) ANOVA where nematicide and nematicide by year interactions were treated as fixed effects of interest and year was treated as a random effect not of scientific interest. For all variables, if nematicide main effects were significant (*P* < 0.05), means were separated using Fisher’s LSD (*P* < 0.05).

## Results and discussion

### Plant-parasitic nematodes and crop yield

**Figure 1:**
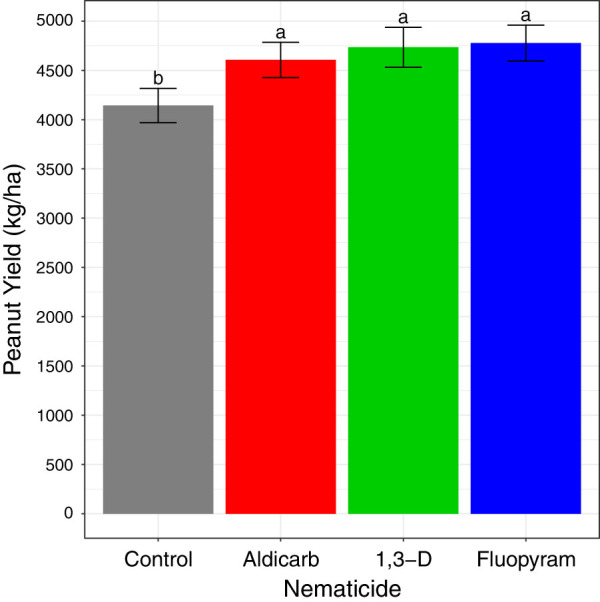
2017 peanut yield as affected by nematicide treatments. Different letters indicate significantly different means based on Fisher’s protected LSD at *α* = 0.05.

Nematicide application did not significantly affect root-knot nematode or total herbivores in any season or year (Table 2). Numerically, 1,3-D reduced root-knot nematode soil abundances in Fall 2017. Root galling was not significantly affected by nematicide treatments in 2017 (mean 14.5% root surface galled) or 2018 (1.3% root surface galled). In 2017, pod galling was significantly greater for fluopyram (15.6% pod surface galled) than control (11.3%), aldicarb (9.4%), or 1,3-D (7.6%). In 2018 pod galling was minimal (0.70% pod surface galled on average) and not affected by treatments. In 2017, application of all nematicides tested increased peanut yield compared with untreated control (Fig. 1). In 2018, nematicide treatments did not significantly affect peanut yield (mean yield 7253 kg/ha).

**Table 2.** Total herbivore and root-knot nematode abundances/100 cm^3^ soil as affected by nematicide application.

	Pi^a^	Pm	Pf
*Herbivores*			
Nematicide			
Control	606	348	1,506
Aldicarb	310	370	1,428
Fluopyram	321	375	1,779
1,3-Dichloropropene	326	350	1,088
ANOVA^b^			
Nematicide (N)	ns	ns	ns
Year × Nematicide	ns	ns	ns
*Root-knot nematodes−2017*			
Nematicide			
Control	30	64	1,755
Aldicarb	26	69	1872
Fluopyram	58	72	2,284
1,3-Dichloropropene	7	33	1,041
ANOVA			
Nematicide (N)	ns	ns	ns
*Root-knot nematodes−2018*			
Nematicide			
Control	725	12	348
Aldicarb	437	11	35
Fluopyram	403	13	281
1,3-Dichloropropene	549	0	26
ANOVA			
Nematicide (N)	ns	ns	ns

**Notes:** Herbivores are displayed for 2017 and 2018 trials combined. Root-knot nematode values are displayed for 2017 and 2018 separately. ^a^Pi, Pm, and Pf are mean values prior to planting, at midseason (62 and 67 days after planting in 2017 and 2018), and at harvest, respectively. ^b^ns represents not significant at *P* > 0.05.

Definite conclusions about the efficacy of the nematicides tested in this trial cannot be made because PRKN pressure was low in 2018, so only one year of data (2017) under meaningful nematode pressure is available. The 2017 results show that all three nematicides-fluopyram, aldicarb, and 1,3-D can effectively increase yield. Despite a lack of statistically significant root-knot nematode management, yield benefits of nematicide in 2017 were likely due to nematode management since 1,3-D has activity almost exclusively against nematodes and no yield benefits of any product were observed in 2018 when nematode pressure was lower. Additionally, numerical trends in PRKN soil abundances suggest that yield benefits were due in part to PRKN control. For fluopyram, some yield increase due to fungal pathogen and insect management cannot be ruled out entirely because fluopyram has activity against fungi and the product used included the insecticide imidacloprid. Similarly, aldicarb has activity against insects. The discrepancy between yield benefits and nematode soil abundances and galling in 2017 can be explained in part by the fact that nematode measurements were made in small plots that did not represent the whole strips on which yield was collected. Average nematode pressure may have been greater in the whole strip than small plots and the large strips may have helped minimize variability, increasing power to detect statistical differences among treatments. In future research, aids to assess nematode damage on a large scale, such as aerial imaging, may be valuable tools for assessment.

From prior research, aldicarb and 1,3-D are known to have efficacy in managing PRKN as both products can help reduce PRKN abundances and increase yield, although inconsistency among years and sites is common (Rodriguez-Kabana and Robertson, 1987; Kinloch and Dickson, 1991; Rodriguez-Kabana et al., 1994a, 1994b; Rodriguez-Kabana et al., 1995; Johnson et al., 1999). Similar to this study, yield responses and ability to detect management of PRKN soil abundances and galling tend to be worse in longer crop rotations and other situations where PRKN infestation is lower (Rodriguez-Kabana and Robertson, 1987; Kinloch and Dickson, 1991; Rodriguez-Kabana et al., 1994a, 1994b; Rodriguez-Kabana et al., 1995; Johnson et al., 1999). Fewer studies on 1,3-D than on aldicarb in peanut have been reported, but in one study, 1,3-D increased yield and managed PRKN both years (Rodriguez-Kabana and Robertson, 1987), whereas in another study, 1,3-D was effective at both reducing PRKN and increasing yield at one site where PRKN infestation was high, but ineffective at another site where PRKN infestation was low (Kinloch and Dickson, 1991). Further research is needed to determine the efficacy of fluopyram, aldicarb, and 1,3-D relative to one another for managing PRKN in peanut production.

### Free-living nematodes

Application of 1,3-D reduced total fungivore soil abundance relative to untreated and relative to fluopyram in midseason and fluopyram in fall (Table 3). Among individual genera of fungivores, *Aphelenchoides* was not significantly affected by nematicide application in any season, but both *Filenchus* and *Aphelenchus* soil abundances were significantly reduced by fumigant 1,3-D application relative to untreated control at midseason only (Table 3). *Filenchus* abundances were also greater for fluopyram than 1,3-D at midseason.

**Table 3.** Fungivores abundances/100 cm^3^ soil as affected by nematicide application for 2017 and 2018 trials combined.

	Pi^a^	Pm^b^		Pf	
*Fungivores*					
Nematicide					
Control	198	159	a	245	ab
Aldicarb	214	82	ab	229	ab
Fluopyram	217	130	a	323	a
1,3-Dichloropropene	203	29	b	168	b
ANOVA^c^					
Nematicide (N)	ns	*		*	
Year × Nematicide	ns	ns		ns	
*Filenchus*					
Nematicide					
Control	42	31	a	58	
Aldicarb	27	19	ab	46	
Fluopyram	37	40	a	65	
1,3-Dichloropropene	23	6	b	46	
ANOVA					
Nematicide (N)	ns	*		ns	
Year × Nematicide	ns	ns		ns	
*Aphelenchus*					
Nematicide					
Control	55	33	a	48	
Aldicarb	52	19	ab	51	
Fluopyram	63	27	ab	63	
1,3-Dichloropropene	66	11	b	23	
ANOVA					
Nematicide (N)	ns	*		ns	
Year × Nematicide	*	ns		ns	
*Aphelenchoides*					
Nematicide					
Control	85	34		135	
Aldicarb	121	20		126	
Fluopyram	104	27		185	
1,3-Dichloropropene	109	11		92	
ANOVA					
Nematicide (N)	ns	ns		ns	
Year × Nematicide	ns	ns		ns	

^a^Pi, Pm, and Pf are mean values prior to planting, at midseason (62 and 67 days after planting in 2017 and 2018), and at harvest, respectively. ^b^Values followed by different letters in the same column for the same variable are significantly different according to Fischer’s LSD at *P* < 0.05. *Represent significant effects at *P* ≤ 0.05; ns represents not significant at *P* > 0.05.

Neither the abundance of total bacterivore nor individual bacterivore genera (*Cephalobus*, *Eucephalobus*, and *Acrobeles*) were significantly affected by nematicide application in any season (Table 4). Similarly, neither total omnivore-predator nor the omnivore *Aporcelaimellus* abundances were significantly affected by nematicide application in any season (Table 5). Genera diversity and trophic guild diversity were also not significantly affected by nematicide treatments in any season. There were an estimated six to eight abundant genera and three to four abundant trophic guilds in the trials based on Hill’s N1 diversity index.

**Table 4.** Bacterivore abundances/100 cm^3^ soil as affected by nematicide application for 2017 and 2018 trials combined.

	Pi^a^	Pm	Pf
*Bacterivores*			
Nematicide			
Control	515	356	360
Aldicarb	508	305	360
Fluopyram	540	337	484
1,3-Dichloropropene	555	351	395
ANOVA^b^			
Nematicide (N)	ns	ns	ns
Year × Nematicide	ns	ns	ns
*Cephalobus*			
Nematicide			
Control	274	123	148
Aldicarb	228	97	153
Fluopyram	257	115	184
1,3-Dichloropropene	261	123	144
ANOVA			
Nematicide (N)	ns	ns	ns
Year × Nematicide	ns	ns	ns
*Eucephalobus*			
Nematicide			
Control	50	56	46
Aldicarb	79	57	47
Fluopyram	63	59	82
1,3-Dichloropropene	94	69	68
ANOVA			
Nematicide (N)	ns	ns	ns
Year × Nematicide	ns	ns	ns
*Acrobeles*			
Nematicide			
Control	72	70	24
Aldicarb	92	66	35
Fluopyram	83	75	36
1,3-Dichloropropene	74	91	44
ANOVA			
Nematicide (N)	ns	ns	ns
Year × Nematicide	ns	ns	ns

**Notes:** ^a^Pi, Pm, and Pf are mean values prior to planting, at midseason (47 and 64 days after planting in 2017 and 2018), and at harvest, respectively; ^b^ns represents not significant at *P* > 0.05.

**Table 5.** Omnivore-predator abundances/100 cm^3^ soil and diversity as affected by nematicide application for 2017 and 2018 trials combined.

	Pi^a^	Pm	Pf
*Omnivore-predators*			
Nematicide			
Control	37	19	33
Aldicarb	50	24	29
Fluopyram	38	11	19
1,3-Dichloropropene	40	27	45
ANOVA^b^			
Nematicide (N)	ns	ns	ns
Year × Nematicide	ns	ns	ns
*Aporcelaimellus*			
Nematicide			
Control	22	9	12
Aldicarb	27	11	15
Fluopyram	16	6	6
1,3-Dichloropropene	16	14	17
ANOVA			
Nematicide (N)	ns	ns	ns
Year × Nematicide	ns	ns	ns
*Hill’s N1 Genera Diversity*			
Nematicide			
Control	6.9	7.7	5.6
Aldicarb	8.8	7.9	5.6
Fluopyram	8.9	7.3	5.6
1,3-Dichloropropene	8.1	5.9	6.2
ANOVA			
Nematicide (N)	ns	ns	ns
Year × Nematicide	ns	ns	ns
*Hill’s N1 Guild Diversity*			
Nematicide			
Control	3.9	4.2	2.9
Aldicarb	4.6	4.3	3.0
Fluopyram	4.5	4.1	2.9
1,3-Dichloropropene	4.2	3.6	3.2
ANOVA			
Nematicide (N)	ns	ns	ns
Year × Nematicide	ns	ns	ns

**Notes:** ^a^Pi, Pm, and Pf are mean values prior to planting, at midseason (47 and 64 days after planting in 2017 and 2018), and at harvest, respectively;

^b^ns represents not significant at *P* >0.05.

Based on these results, the fumigant 1,3-D was detrimental to free-living nematodes, namely fungivores, but the non-fumigants aldicarb or fluopyram were no more detrimental than not applying nematicide. In general, nematicide effects on free-living nematodes in this study were less than that observed in other studies. Greater fumigant than non-fumigant impacts on the nematode community is consistent with most previous research (Sanchez-Moreno et al., 2010; Timper et al., 2012; Watson and Desaeger, 2019). The lack of impact of aldicarb application on free-living nematodes is not consistent with prior literature (Smolik, 1983; Grabau and Chen, 2016; Grabau et al., 2018). Prior aldicarb studies were conducted on heavier soils in Midwestern corn or soybean systems which may partially account for the greater non-target effects in other studies (Smolik, 1983; Grabau and Chen, 2016; Grabau et al., 2018). Additionally, to investigate specific hypotheses, two studies on aldicarb used a higher than labeled rate at 2.94 kg/ha (Grabau and Chen, 2016; Grabau et al., 2018), whereas this study used the current labeled rate of 1.18 kg a.i./ha. Smolik (1983) observed non-target effects on microbial-feeding nematodes and Dorylaimids (primarily omnivores) at both a similar rate to this study (1.12 kg a.i./ha) and a higher rate (2.24 kg a.i./ha), so aldicarb application rate does not account for differences between the two studies.

Limited research has been conducted on fluopyram influence on free-living nematodes, but a study in Florida strawberry (*Fragaria* × *ananassa*) production found that fluopyram applied at 0.48 or 0.50 kg a.i/ha − approximately double the rate in this study − had minimal influence on any group of free-living nematodes, which was similar to this study (Watson and Desaeger, 2019). In contrast, in Florida turfgrass, fluopyram had a substantial and consistent negative effect on all groups of free-living nematodes (Waldo et al., 2019), but practices differed from this study, based on the differing plant systems, which may account for the differences between the two studies. In Waldo et al.’s (2019) study, nematode populations were monitored at a more shallow depth (7 vs 30 cm), nematicide applications covered a larger area (broadcast vs in-furrow), nematicide applications were repeated more times (4 applications vs 1), and total fluopyram applied was greater (2.0 kg a.i./ha compared with 0.24 kg a.i./ha) than in this study.

Fungivores were more sensitive to 1,3-D nematicide application than bacterivores or omnivore-predators. In general, fumigants, including 1,3-D, tend to affect a broad spectrum of free-living nematodes (Sanchez-Moreno et al., 2010; Timper et al., 2012; Watson and Desaeger, 2019). In other studies, the trophic groups affected by 1,3-D varied somewhat, but there were no consistent trends among studies that a particular group was more sensitive to 1,3-D application than any other group (Sanchez-Moreno et al., 2010; Timper et al., 2012; Watson and Desaeger, 2019). Fumigation affected a more narrow spectrum of free-living nematodes in this study than others and this may be because it was applied at a lower rate than in other studies based on standard practices and economic constraints in the respective crops (Sanchez-Moreno et al., 2010; Watson and Desaeger, 2019) or scientific choices (Timper et al., 2012). Specifically, in this study, 1,3-D was applied at 38.6 kg a.i./ha before planting. Sanchez-Moreno et al. (2010) applied 1,3-D at 100 kg a.i./ha and mixed with chloropicrin at 182 kg a.i./ha. Watson and Desaeger (2019) applied 1,3-D at 125 kg a.i./ha and mixed with chloropicrin at 190 kg a.i./ha. Timper et al. (2012) applied 1,3-D at 66 kg a.i/ha before planting and aldicarb at 1.0 kg a.i./ha at planting.

Impacts of 1,3-D were also relatively persistent, considering fungivore abundances did not rebound by the end of the growing season after a single application of the product, which is consistent with other studies (Sanchez-Moreno et al., 2010; Watson and Desaeger, 2019). Timper et al. (2012) did not measure impacts on free-living nematode populations at harvest, but in the spring following 1,3-D with aldicarb application, there were no residual effects on free-living nematode abundances despite applying nematicide repeatedly in the same plots for three years. Many growers are likely to apply nematicides repeatedly on a yearly basis, so research testing the impacts of repeated application of various nematicides on PRKN management and free-living nematodes in peanut production would be of practical importance.

Individual free-living nematode genera did vary in their sensitivity to nematicide application, but differences were split almost entirely among, and not within, trophic groups. Fungivore genera were more sensitive than bacterivore and omnivore-predator genera, but there were minimal meaningful differences among genera within each trophic group. Additionally, total trophic group abundances tended to be more strongly influenced by nematicide application than individual genera, likely due to the greater abundances in the total trophic group helping to minimize variation and magnify differences. While the results from this study suggest total trophic group abundance is a more important and sensitive measure than individual genera, other studies have found that individual genera vary in their sensitivity to specific agricultural disturbances (Zhao and Neher, 2013). Most other research studies on the influence of nematicide application on the nematode community report on trophic groups or indices not individual genera (Sanchez-Moreno et al., 2010; Timper et al., 2012; Watson and Desaeger, 2019; Waldo et al., 2019), so additional research to determine if individual genera vary in their sensitivity to nematicide application should be considered.

## Conclusion

In summary, fluopyram, aldicarb, and 1,3-D nematicides can help reduce yield losses from PRKN, but further years of research with substantial PRKN pressure is needed to validate the efficacy of these products relative to each other. In peanut production, at commercial rates, single applications of nematicides had relatively little effect on free-living nematodes. The fumigant 1,3-D was somewhat detrimental to fungivore abundances, but the non-fumigant nematicides fluopyram or aldicarb were not detrimental to abundances of any free-living nematodes.
